# Use of Artificial Intelligence Chatbots in Interpretation of Pathology Reports

**DOI:** 10.1001/jamanetworkopen.2024.12767

**Published:** 2024-05-22

**Authors:** Eric Steimetz, Jeremy Minkowitz, Elmer C. Gabutan, Joan Ngichabe, Hagar Attia, Mordechai Hershkop, Fatih Ozay, Matthew G. Hanna, Raavi Gupta

**Affiliations:** 1 Department of Pathology, SUNY (State University of New York) Downstate Medical Center, Brooklyn; 2 Lake Erie College of Osteopathic Medicine, Elmira, New York; 3 Department of Pathology, Memorial Sloan Kettering Cancer Center, New York, New York

## Abstract

**Importance:**

Anatomic pathology reports are an essential part of health care, containing vital diagnostic and prognostic information. Currently, most patients have access to their test results online. However, the reports are complex and are generally incomprehensible to laypeople. Artificial intelligence chatbots could potentially simplify pathology reports.

**Objective:**

To evaluate the ability of large language model chatbots to accurately explain pathology reports to patients.

**Design, Setting, and Participants:**

This cross-sectional study used 1134 pathology reports from January 1, 2018, to May 31, 2023, from a multispecialty hospital in Brooklyn, New York. A new chat was started for each report, and both chatbots (Bard [Google Inc], hereinafter chatbot 1; GPT-4 [OpenAI], hereinafter chatbot 2) were asked in sequential prompts to explain the reports in simple terms and identify key information. Chatbot responses were generated between June 1 and August 31, 2023. The mean readability scores of the original and simplified reports were compared. Two reviewers independently screened and flagged reports with potential errors. Three pathologists reviewed the flagged reports and categorized them as medically correct, partially medically correct, or medically incorrect; they also recorded any instances of hallucinations.

**Main Outcomes and Measures:**

Outcomes included improved mean readability scores and a medically accurate interpretation.

**Results:**

For the 1134 reports included, the Flesch-Kincaid grade level decreased from a mean of 13.19 (95% CI, 12.98-13.41) to 8.17 (95% CI, 8.08-8.25; *t* = 45.29; *P* < .001) by chatbot 1 and 7.45 (95% CI, 7.35-7.54; *t* = 49.69; *P* < .001) by chatbot 2. The Flesch Reading Ease score was increased from a mean of 10.32 (95% CI, 8.69-11.96) to 61.32 (95% CI, 60.80-61.84; *t* = −63.19; *P* < .001) by chatbot 1 and 70.80 (95% CI, 70.32-71.28; *t* = −74.61; *P* < .001) by chatbot 2. Chatbot 1 interpreted 993 reports (87.57%) correctly, 102 (8.99%) partially correctly, and 39 (3.44%) incorrectly; chatbot 2 interpreted 1105 reports (97.44%) correctly, 24 (2.12%) partially correctly, and 5 (0.44%) incorrectly. Chatbot 1 had 32 instances of hallucinations (2.82%), while chatbot 2 had 3 (0.26%).

**Conclusions and Relevance:**

The findings of this cross-sectional study suggest that artificial intelligence chatbots were able to simplify pathology reports. However, some inaccuracies and hallucinations occurred. Simplified reports should be reviewed by clinicians before distribution to patients.

## Key Points

**Question:**

Can artificial intelligence chatbots accurately simplify pathology reports so that patients can easily understand them?

**Findings:**

In this cross-sectional study of 1134 pathology reports, 2 chatbots significantly decreased the reading grade level of pathology reports while interpreting most reports correctly. However, some reports contained significant errors or hallucinations.

**Meaning:**

These findings suggest that chatbots have the potential to explain pathology reports to patients and extrapolate pertinent details; however, they are not flawless and should not be used without a review by a health care professional.

## Teaser

This cross-sectional study investigates the ability of artificial intelligence chatbots to accurately simplify anatomic pathology reports for patients and identify key elements of the reports that are pertinent to patient care.

## Introduction

Every day, thousands of anatomic pathology specimens are processed across the US. On reviewing the slides and, potentially, ancillary test results, the pathologist prepares a report with the diagnosis. Depending on the procedure and complexity of the case, the report often contains additional information or comments, and often in cancer cases, prognostic information and molecular therapeutic targets that directly affect patient management. Despite their ubiquity and importance, the reports are written in difficult language that is generally beyond the comprehension of laypeople. Moreover, the reports have grown increasingly complex and lengthy in recent years.^1^ In contrast to laboratory values, anatomic pathology reports are not standardized and often contain nuanced phrasing that even experienced clinicians may interpret incorrectly.^2^ With the advent of electronic portals, patients have unfettered access to their reports. However, the complexity of the reports presents a major barrier to large-scale adoption of patient portals.^3^

Recent advancements in artificial intelligence (AI) have given rise to large language models (LLMs), which are probabilistic natural language processing systems trained on copious quantities of data. Large language model chatbots are generative AI applications that produce output in response to input in a conversational manner.^4^ An initial hypothesis was that the technology could be used in clinical decision support applications. However, several studies^4,5,6^ have shown that, in its current form, the technology is too error prone and limited to be efficacious in the clinical setting. Nonetheless, previous studies^7,8^ have shown that chatbots can communicate health information effectively. In fact, 1 study^7^ found the responses given by chatbots to patient questions to be of better quality and to appear to exhibit more empathy than some responses given by physicians. Thus far, the applicability of chatbots in the diagnostic setting remains largely unexplored. In this study, we investigate the ability of chatbots to accurately simplify anatomic pathology reports for patients and identify key elements of the reports that are pertinent to patient care.

## Methods

This cross-sectional study was deemed exempt from review and informed consent by the SUNY (State University of New York) Downstate Health Sciences University Institutional Review Board. The study followed the Strengthening the Reporting of Observational Studies in Epidemiology (STROBE) reporting guideline.

### Data Collection

We retrieved 1134 pathology reports from specimens processed between January 1, 2018, and May 31, 2023, in a teaching hospital in Brooklyn, New York. The reports were of varying length and complexity, written by different pathologists, and addressing a variety of different procedures, organ systems, and conditions (Table 1). The reports included many specimens from ordinary procedures such as appendectomies, Papanicolaou smears, and skin, breast, prostate, and colon biopsies. The diagnoses cover many conditions, such as lipoma, appendicitis, prostatic adenocarcinoma, invasive mammary carcinoma, colonic adenocarcinoma, and melanoma. Comments, notes, addendums, and synoptic reports (where applicable) were often part of the report. Some reports consisted of multipart resections, while others consisted of a single biopsy. The text of the reports was not edited for clarity. Potentially identifying information, such as dates, references to specimen accession numbers, and the names of the pathologists and clinicians, were anonymized. Care was taken to avoid including multiple reports with identical text, such as cytology reports, which have standardized sign-out language.

**Table 1.** Reports Categorized by Organ System and Severity

Organ system	Biopsy finding, No. (%) (N = 1134)						
	Normal	Benign	Atypical and/or suspicious	Precancerous	Malignant	Nondiagnostic	All
Bone and soft tissue	0	68 (6.00)	5 (0.44)	1 (0.09)	26 (2.29)	0	100 (8.82)
Breast	4 (0.35)	47 (4.14)	8 (0.71)	16 (1.41)	32 (2.82)	0	107 (9.44)
Cytology	8 (0.71)	42 (3.70)	26 (2.29)	10 (0.88)	9 (0.79)	5 (0.44)	100 (8.82)
Dermatology	0	78 (6.88)	1 (0.09)	14 (1.23)	11 (0.97)	0	104 (9.17)
ENT	16 (1.41)	61 (5.38)	3 (0.26)	10 (0.88)	20 (1.76)	0	110 (9.70)
GI	25 (2.20)	58 (5.11)	9 (0.79)	34 (3.00)	28 (2.47)	0	154 (13.58)
GU	10 (0.88)	55 (4.85)	8 (0.71)	5 (0.44)	42 (3.70)	0	120 (10.58)
GYN	22 (1.94)	54 (4.76)	6 (0.53)	28 (2.47)	19 (1.68)	3 (0.26)	132 (11.64)
Hepatobiliary	1 (0.09)	40 (3.53)	7 (0.62)	6 (0.53)	52 (4.59)	0	106 (9.35)
Pulmonary	10 (0.88)	44 (3.88)	5 (0.44)	0	42 (3.70)	0	101 (8.91)
All	96 (8.47)	547 (48.24)	78 (6.88)	124 (10.93)	281 (24.78)	8 (0.71)	1134 (100)

Abbreviations: ENT, otorhinolaryngologic; GI, gastrointestinal; GU, genitourinary; GYN, gynecologic.

Two reviewers (E.S. and F.O.) independently categorized findings of each report as normal, benign, atypical and/or suspicious, precancerous, or malignant. In case of disagreement, a third pathologist (R.G.) functioned as a tiebreaker. During the review process, several cases were found to be nondiagnostic and were classified as such (Table 1).

### Study Design

Two chatbots were used in this study: Bard (Google Inc; versions June 7, 2023, and July 13, 2023), referred to hereinafter as chatbot 1, and GPT-4 (OpenAI; version May 24, 2023), referred to hereinafter as chatbot 2. The models were queried between June 1 and August 31, 2023.

The pathology reports were input to the chatbots, which were asked, sequentially, to simplify the report; to classify findings on the spectrum of normal to malignant; and to denote the pathologic stage of the tumor (Table 2). To minimize bias, a new chat thread was started for each report, and the last question was asked in all cases, regardless of the diagnosis. The chatbot response to each prompt was recorded; eFigures 1 and 2 in Supplement 1 depict a sample exchange.

**Table 2.** Text of Questions Input to Chatbots

Question No.	Question text
1	Please explain the following pathology report to someone without any high school education. Be concise: “Pathology report”
2	Respond in one word. Is the diagnosis benign or malignant?
3	Choose one word to characterize the report. Your choices are normal, benign, atypical, precancerous, malignant.
4	What is the pathologic stage of the tumor?

A commercial web-based tool (readable.com) was used to assess the readability metrics of the simplified (ie, the response to question 1) and original reports. The word count and findings of 2 widely used readability formulas, the Flesch Reading Ease (FRE) and Flesch-Kincaid grade level (FKGL), were recorded.^9^

In addition to readability, the accuracy of the simplified report was assessed. Two reviewers (including E.S., J.M., E.C.G., J.N., H.A., and F.O.) independently screened the reports for errors. Three pathologists (E.S., M.G.H., and R.G.) evaluated the flagged reports and categorized them as medically correct, partially medically correct, or medically incorrect. Medically correct indicated that the simplified report contained no errors, and the information was medically sound; partially medically correct, the simplified report contained at least 1 erroneous statement or explanation, but not one that would drastically alter the medical management of the disease or condition (eg, misstating the precise size of the tumor or miscounting the number of positive lymph nodes); and medically incorrect, the simplified report contained a significant error that would drastically change the management of the patient (eg, stating that cancer was present in a benign specimen or an incorrect hormonal status of a breast tumor). The reviewers also recorded any instances of hallucinations, which are fabricated statements or explanations.

### Statistical Analysis

The mean readability scores of the original and simplified reports were compared using paired-samples *t* tests. The mean readability scores of the simplified reports generated by both chatbots were compared using a 2-tailed independent-samples *t* test. Statistical analysis was conducted using the open source SciPy Python package, version 1.9.3 (SciPy Community). Two-sided *P* < .05 indicated statistical significance.

## Results

The mean FKGL of the original 1134 reports was 13.19 (95% CI, 12.98-13.41), the mean FRE score was 10.32 (95% CI, 8.69-11.96), and the mean word count was 80.65 (95% CI, 73.34-87.96). A total of 1068 reports (94.18%) were at or above an FKGL of 9.00. The mean FKGL of reports simplified by chatbot 1 was 8.17 (95% CI, 8.08-8.25), the mean FRE score was 61.32 (95% CI, 60.80-61.84), and the mean word count was 192.38 (95% CI, 188.44-196.32); a total of 314 reports (27.69%) were at or above an FKGL of 9.00. The mean FKGL of reports simplified by chatbot 2 was 7.45 (95% CI, 7.35-7.54), the mean FRE score was 70.80 (95% CI, 70.32-71.28), and the mean word count was 145.43 (95% CI, 141.04-149.81); a total of 197 reports (17.37%) were at or above an FKGL of 9.00.

The results of a paired *t* test showed that the simplified reports generated by both chatbots significantly decreased the FKGL (*t* = 45.29 [chatbot 1] and *t* = 49.69 [chatbot 2]; *P* < .001 for both) and increased the FRE score (*t* = −63.19 [chatbot 1] and *t* = −74.61 [chatbot 2]; *P* < .001 for both). An independent *t* test showed a significant difference in mean readability scores between the simplified reports generated by the 2 chatbots for both the FKGL and FRE score (*t* = −26.32 and *t* = 10.92, respectively; *P* < .001 for both).

**Figure 1.**
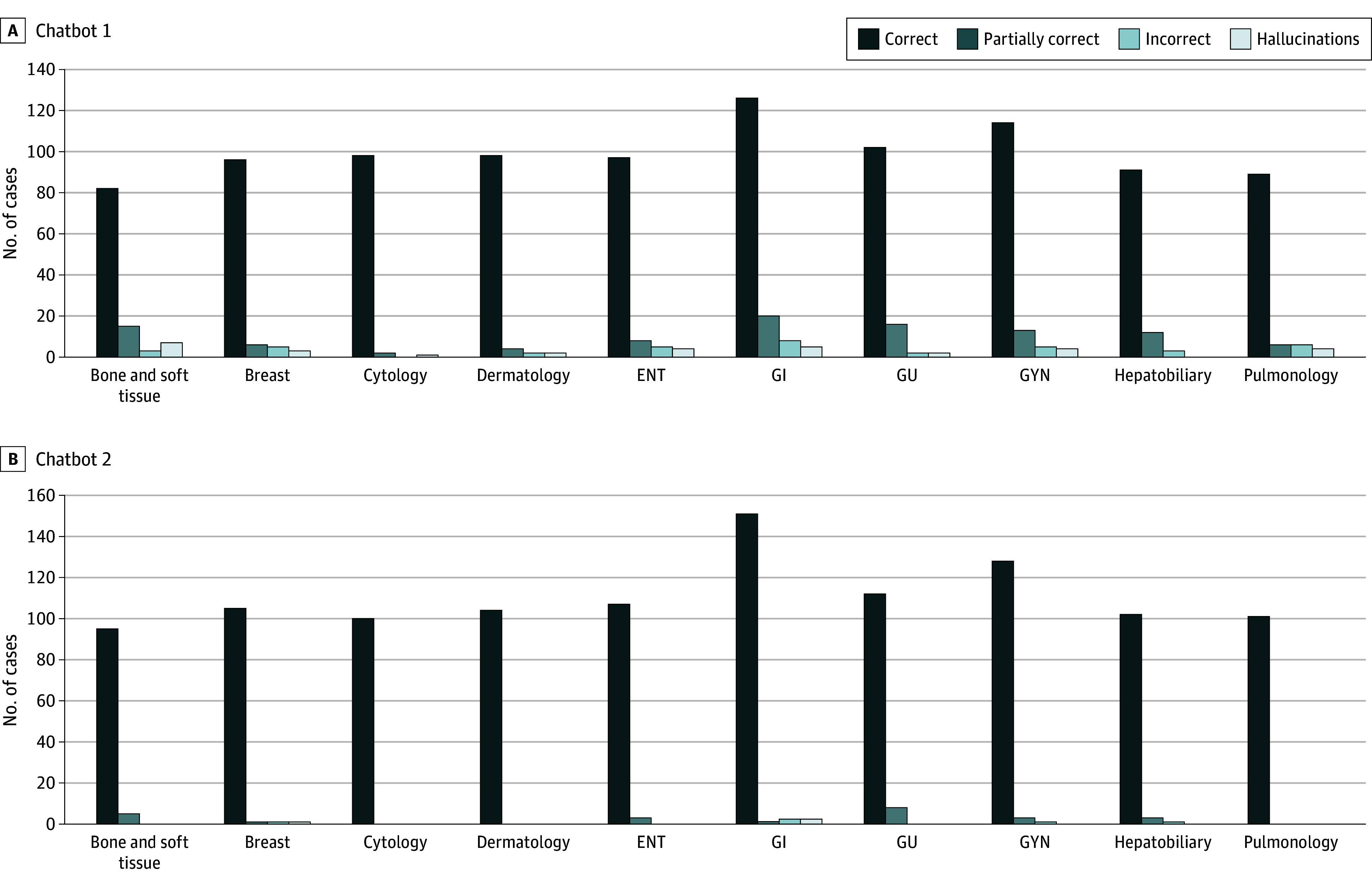
Medical Accuracy of Chatbot Interpretation of Simplified Reports by Subspecialty ENT indicates otorhinolaryngologic; GI, gastrointestinal; GU, genitourinary; and GYN, gynecologic.

Overall, both chatbots interpreted most reports correctly (Figure 1). For chatbot 1, 993 reports (87.57%) were medically correct, 102 (8.99%) were partially correct, and 39 (3.44%) were incorrect; 32 reports (2.82%) contained hallucinations. For chatbot 2, 1105 reports (97.44%) were medically correct, 24 (2.12%) were partially correct, and 5 (0.44%) were incorrect; 3 reports (0.26%) contained hallucinations.

The most common error made by both chatbots was assuming that a resection specimen without lymph nodes (pNx) implied that the lymph nodes status was negative (pN0). Common hallucinations included statements about patients, such as their well-being after the procedure, and confabulatory explanations of unfamiliar terms. Select reports are shown in Table 3.

**Table 3.** Simplified Chatbot Reports With Accuracy Grading

Original report	Simplified report^a^	Medically accurate	Hallucinations
**Chatbot 1** ^b^				
Left mastoid mass; excision: - Spindle cell lipoma	Sure. A spindle-cell lipoma is a type of benign (noncancerous) tumor that is made up of fat cells and spindle-shaped cells. It is most commonly found in the neck, shoulder, or upper back of elderly men. In this case, the patient had a spindle-cell lipoma in their left mastoid bone (the bone behind the ear). The tumor was removed surgically and the patient recovered well. Here is a more concise explanation: A noncancerous tumor was removed from the patient’s left mastoid bone. The patient is doing well.	Yes	Yes
1. Skin, left posterior arm, shave biopsy: - Seborrheic keratosis, rubbed 2. Skin, right posterior ear, shave biopsy: - Seborrheic keratosis	Sure, I can help you with that. The pathology report you provided says that you had 2 skin biopsies done, 1 on your left posterior arm and 1 on your right posterior ear. The results of the biopsies showed that you have seborrheic keratosis, which is a common, benign skin growth. Seborrheic keratosis is often described as a “stuck-on” growth that can be brown, black, or tan. It can be slightly raised or flat, and it can sometimes feel greasy. Seborrheic keratosis is usually harmless, but it can sometimes be removed if it is bothersome or unsightly. The word “rubbed” in the pathology report means that the biopsy specimen was rubbed to remove some of the excess keratin, which is the hard, outer layer of the skin. This was done to make it easier to see the underlying cells of the skin growth. I hope this explanation is helpful. Please let me know if you have any other questions.	Yes	Yes
Tonsil, right, biopsy: - Squamous papilloma; see note Note: Immunohistochemical stains for Ki67 and p16 were reviewed Ki67 shows staining within basal cells; p16 is negative	Sure, here is a concise explanation of the pathology report: The tissue sample from your right tonsil is a benign growth called a squamous papilloma. This growth is made up of cells that normally line the tonsils. The Ki67 stain shows that the cells in the growth are dividing rapidly, which is a sign of a benign growth. The p16 stain is negative, which means that the growth is not caused by the human papillomavirus (HPV). In other words, the growth is harmless and does not need to be treated. However, your doctor may want to keep an eye on it to make sure it does not grow or spread. Here is a more detailed explanation of the terms used in the report: Squamous papilloma: A benign growth that is made up of cells that normally line the skin or mucous membranes. Ki67: A protein that is present in cells that are dividing rapidly. p16: A protein that is produced by cells that are infected with HPV. Negative: The absence of a particular protein or substance. I hope this explanation is helpful. Please let me know if you have any other questions.	Partially	No
**Chatbot 2** ^c^				
Vagina, apex, biopsy: - Fragments of benign squamous epithelium - No dysplasia seen	Sure, I’ll try to make it as simple as possible. This report is about a small sample of tissue that was taken from the top part of your vagina for testing. The test shows that the skinlike cells in the sample are healthy, not harmful. They didn’t find any abnormal, potentially precancerous cells. So, the results are good.	Yes	No
Urinary bladder; transurethral resection of bladder tumor (TURBT): - High grade urothelial carcinoma (see comment) - No evidence of invasion - No muscularis propria identified	Sure, I’ll break that down in simple terms: The doctor did a surgery to remove a growth from the bladder, which is the bag-like part of your body that stores pee. This surgery was done by going up through the tube where you pee, so there were no big cuts on your body. The growth that was removed was tested, and it turns out to be a kind of bad cancer. But the good news is, the cancer doesn’t seem to have spread to other parts of the bladder, especially not the muscles. They didn’t find any deep muscle layer of the bladder in the sample they took out, which means the cancer likely didn’t get that deep. This report is mostly good news: the cancer was caught early and hasn’t spread deep into your bladder.	Partially	No
Esophagus, distal, biopsy: - Squamous epithelium with marked acute, chronic inflammation, ulcer and cytological features consistent with herpes simplex virus infection - Fungal organisms morphologically compatible with *Candida* infection are also identified	This report says they took a small sample from the lower part of your food pipe, which is called the esophagus. They found 2 problems: 1. First, they found signs of a sore, and some bad irritation which usually comes from a kind of virus called herpes simplex. This is not typically the same kind that causes cold sores or genital herpes. It’s just the name of the virus. 2. Second, they found a type of fungus called *Candida*. This is similar to a yeast infection which people can get in different parts of their body. Both of these things could be causing discomfort or other symptoms you might be feeling. Your doctor will likely suggest some treatments for these problems.	No	No

^a^ The precise output given by the chatbot for question 1 (from Table 2). White space has been eliminated to condense the text.

^b^ Versions June 7, 2023, and July 13, 2023 (Google Inc).

^c^ Version May 24, 2023 (OpenAI).

### Questions 2 to 4

#### Benign vs Malignant

**Figure 2.**
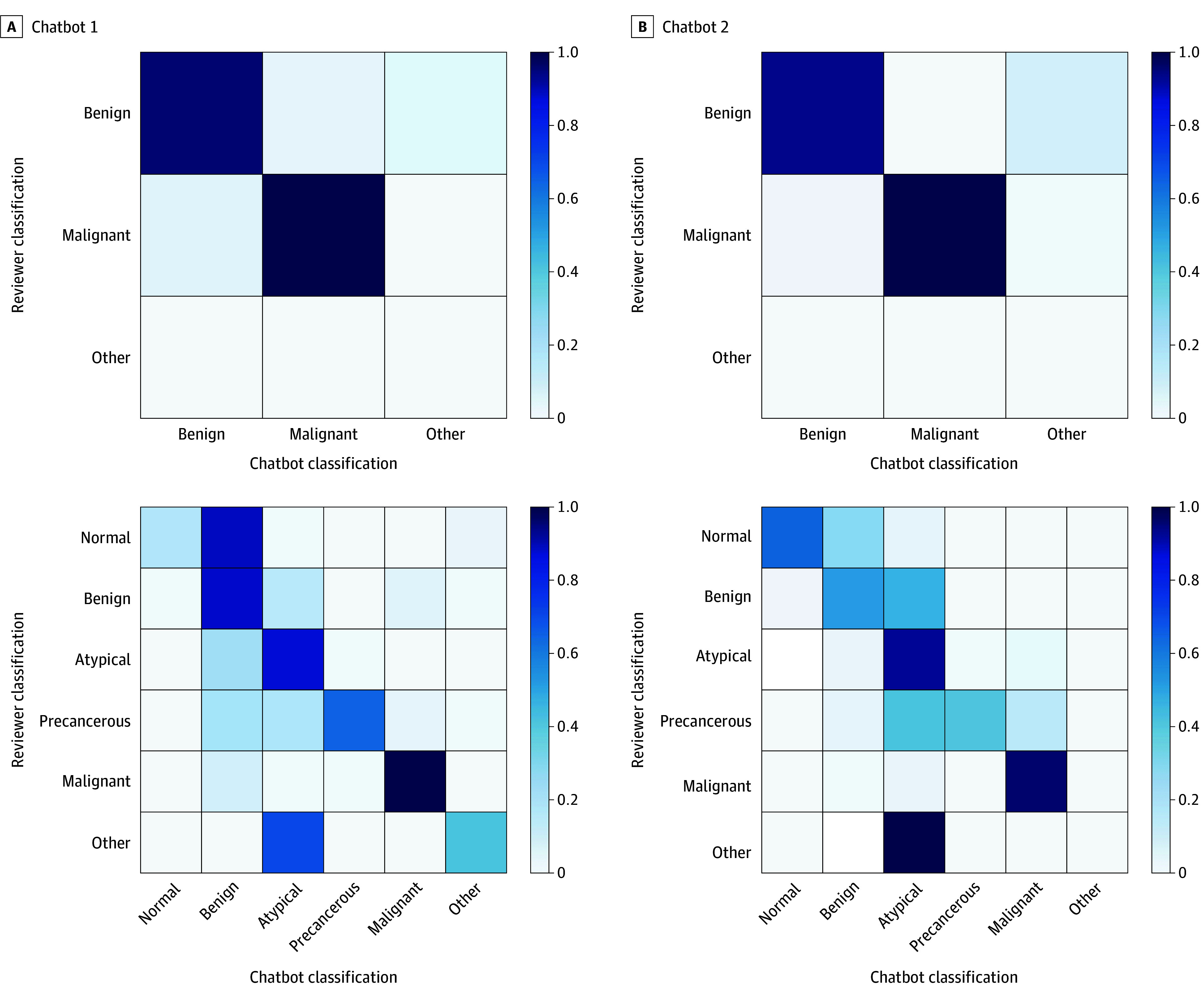
Confusion Matrix for Chatbot Diagnosis Classification Shading indicates correlation between the expected and given value. The darkest hue (1.0) represents a perfect correlation.

Findings in 924 reports were classified by the reviewers as either benign (including normal) or malignant. Of those, 848 (91.77%) of chatbot 1 responses and 857 (92.75%) of chatbot 2 responses were correct (Figure 2).

#### Classifying the Reports on the Normal to Malignant Spectrum 

In all 1134 reports, chatbot 1 responded with a 1-word answer in 28 (2.47%) reports and with 1 of the answer choices given in 1125 reports (99.21%). Chatbot 2 responded with a 1-word answer and with 1 of the answer choices (normal, benign, atypical and/or suspicious, precancerous, or malignant) in all but 1 report (Figure 2).

#### Pathologic Stage 

Of the 1134 reports, 97 (8.55%) contained a pathologic tumor stage. Of those, chatbot 1 responded with the correct stage in 89 cases (91.75%) and chatbot 2 responded with the correct stage in 93 cases (95.88%). Inappropriate stage (ie, providing a stage although nonexistent) was provided by chatbot 1 in 122 cases (10.76% of all reports) and by chatbot 2 in 5 cases (0.44% of all reports).

### Qualitative Findings

The reviewers believed that the responses given by chatbot 2 were better and more comprehensive, while the responses given by chatbot 1 were wordy and less helpful. This finding is supported by the statistically significant differences in mean readability scores and word count. The difference became more pronounced when comparing the performance of the chatbots for other metrics, such as medical accuracy.

## Discussion

To our knowledge, this is the first cross-sectional study to investigate the use of generative AI chatbots as a tool to simplify pathology reports and make them more accessible to patients. The first important study to show the potential use of chatbots in health care was published by Ayers et al,^7^ who reported that chatbots responded to informal patient questions posted on an online forum. Chatbot responses were of fair quality and appeared to exhibit more empathy than some physicians’ answers. Other studies have investigated the ability of chatbots to respond to physician questions and facilitate clinical decision-making, with mixed results.^5,6,10^ While the findings reported by Ayers et al^7^ suggest that chatbots may supplant physicians in some tasks, our findings suggest that they could augment physicians and serve patients by simplifying complex medical information and responding to a limited set of potential follow-up questions. Incorporating these inexpensive technology solutions in clinical practice could reduce disparities and be especially helpful to patients from socioeconomically disadvantaged backgrounds, who tend to have lower health literacy.^11^ Clinicians have a responsibility to convey test results to patients and ensure that they have the requisite knowledge to follow through with treatment. Studies have linked patients’ involvement with their care to health outcomes.^12^ However, it is impossible for patients to participate in the decision-making process without fully understanding their results.^13^ The integration and implementation of sophisticated chatbots into the field of pathology can potentially revolutionize how pathology reports are perceived and understood and allow patients to make informed decisions. Furthermore, as health care professionals increasingly recognize the importance of optimizing patient-physician communication, these models can empower patients by offering immediate interpretation of their reports. This would eliminate the often-anxious wait for their follow-up appointment, leading to improved health care outcomes and use of resources.^14^ Simplified reports may benefit midlevel practitioners and enhance the educational experience of medical students who are less familiar with the highly technical terms often found in pathology reports. Notably, the structured nature of the College of American Pathologists synoptic protocols presents a unique opportunity for these models to easily autogenerate explanatory notes based on the selected fields.

Simplified pathology reports have the potential to make health care more accessible to millions of patients and reduce disparities among those with low health literacy levels. Of note, the grade level of the simplified reports is markedly lower than that of most online patient educational material, which often is written for those with an 11th grade educational level or higher.^15,16,17^

Another potential use of the technology is working in the background to streamline physician workflow. The ability of chatbots to correctly categorize reports into benign, premalignant, and malignant categories can allow for triaging of reports and determining which should be given priority by clinicians, especially in large medical centers with high patient volumes. Results of cases that are in the benign and normal categories could be released to patients without having to schedule a follow-up appointment to discuss the results.

Fine-tuning, which is the process of further training a model on a dataset of the correct answers to a specific question or task, has been shown to enhance the model’s performance in several studies.^4^ It is plausible that training a chatbot on such a dataset could improve its accuracy and reduce the instances of medically incorrect statements or hallucinations. Currently, users can fine-tune chatbot 2 through an application programming interface, but not chatbot 1. Evaluating the capabilities of models that are fine-tuned to explain and classify pathology reports should be a focus of future research.

Presently, the biggest barriers to wide adoption of the technology are hallucinations and seemingly true statements that are factually incorrect. This difficulty of chatbots with quantitative reasoning has been extensively documented in the literature.^18,19^ Although some fact-checking solutions have been proposed,^20,21^ they are unlikely to be useful in situations where facts are nuanced or not readily available (eg, the well-being of a patient after surgery).

A few conditions must be met to deploy such models in clinical settings. We note that the proper ethical considerations, patient privacy, and regulatory compliance must be met. Sharing personal health information with chatbots may violate patient confidentiality.^22^ Critically, the issue of accuracy and hallucinations poses a significant hurdle to the clinical deployment of chatbots that answer patient questions about their pathology report, as it is not possible to anticipate all questions and evaluate every potential response. It is possible that fine-tuned models would perform significantly better and should be a focus of further studies. Until a proper solution is developed and tested, patients should not blindly rely on a response provided by an AI chatbot. Rather, the output should first be reviewed by a health care professional to ensure the response is medically sound.

### Limitations

This study has some limitations. First, the reports were sourced from a single institution. Because pathology reports are largely not standardized, varying report structure or wordings might be interpreted differently by the models, potentially limiting the generalizability of our findings. Second, chatbots are probabilistic in nature and could change their output based on different inputs. The way the questions were phrased, and their order, could have influenced the responses. Additionally, using an evaluation framework that is based on measuring accuracy and hallucinations alone may not adequately capture all the subcomponents of an ideal patient-friendly report, such as clarity, completeness, and empathy. Last, determining the proper category of a given diagnosis can be challenging in some instances. For example, cervical low-grade squamous intraepithelial lesions have a significantly lower malignant potential than colonic polyps with high-grade dysplasia, yet both are considered precancerous. There are many conditions whose proper category is a matter of scientific debate.

## Conclusions

The findings of this cross-sectional study suggest that artificial intelligence chatbots can simplify pathology reports for patients and identify key details that are relevant for patient management. However, their interpretation should be used judiciously, as they are not without flaws. Developing fact-checking solutions is necessary before integrating these tools in the health care setting.

## Notes

Supplement 1. **eFigure 1.** Sample Exchange for Chatbot 1 **eFigure 2.** Sample Exchange for Chatbot 2

Supplement 2.
Data Sharing Statement
