# Supplementary material for: Use of Artificial Intelligence Chatbots in Interpretation of Pathology Reports
Source: JAMA Netw Open. 2024 May 22;7(5):e2412767. doi: 10.1001/jamanetworkopen.2024.12767 (PMC11112436; doi:10.1001/jamanetworkopen.2024.12767)
Supplement: Supplement 1. — eFigure 1. Sample Exchange for Chatbot 1 eFigure 2. Sample Exchange for Chatbot 2 [file jamanetwopen-e2412767-s001.pdf]

Supplementary Online Content

Steimetz E, Minkowitz J, Gabutan EC, et al. Use of artificial intelligence chatbots in interpretation of pathology reports. *JAMA Netw Open*. 2024;7(5):e2412767. doi:10.1001/jamanetworkopen.2024.12767

eFigure 1. Sample Exchange for Chatbot 1

eFigure 2. Sample Exchange for Chatbot 2

This supplementary material has been provided by the authors to give readers additional information about their work.

© 2024 Steimetz E et al. *JAMA Network Open*.

eFigure 1. Sample Exchange for Chatbot 1

Please explain the following pathology report to someone without any high school education. Be concise:
"1. Prostate, left apex, biopsy:
- Benign prostatic tissue.
2. Prostate, left mid, biopsy: ...

Sure, here is a concise explanation of the pathology report that someone without any high school education can understand:

The pathology report shows that the tissue samples taken from your prostate gland are mostly normal. However, there is a small area of atypical cells in one of the samples from both the left and right mid-prostate. This means that the cells in these areas do not look completely normal, but they are not cancerous either. The presence of these atypical cells may be a sign of early prostate cancer, but more testing is needed to confirm this.

The following are some of the possible reasons why the pathologist found atypical cells in your prostate tissue:

- Inflammation: Inflammation can cause changes in the appearance of cells, which can make them look atypical.
- Prostate hyperplasia: Prostate hyperplasia is a noncancerous condition that causes the prostate gland to enlarge. This can also lead to changes in the appearance of cells.
- Prostate cancer: Prostate cancer is a rare form of cancer that starts in the prostate gland. Atypical cells are one of the early signs of prostate cancer.

If you have any questions about the pathology report, please talk to your doctor.

I hope this explanation is helpful. Please let me know if you have any other questions.

Respond in one word. Is the diagnosis benign or malignant?

Benign. The pathology report states that the tissue samples are mostly benign (normal). The presence of atypical cells may be a sign of early prostate cancer, but more testing is needed to confirm this.

Choose one word to characterize the report. Your choices are normal, benign, atypical, pre-cancerous, malignant.

I would use the word **atypical** to characterize the report. This is because the report states that there is a small area of atypical cells in one of the samples from the left and right mid-prostate. This means that the cells in these areas do not look completely normal, but they are not cancerous either. The presence of these atypical cells may be a sign of early prostate cancer, but more testing is needed to confirm this. The other choices are not accurate for this report. The report does not state that the tissue samples are normal, benign, pre-cancerous, or malignant.

What is the pathologic stage of the tumor?

The pathologic stage of the tumor cannot be determined from the pathology report provided. The report only states that there is a small area of atypical cells in one of the samples from the left and right mid-prostate. This does not mean that the tumor is at a specific stage. To determine the pathologic stage of the tumor, a pathologist would need to examine the entire tumor. This can only be done after the tumor has been removed from the body.

The pathologic stage of the tumor is important because it helps to determine the best course of treatment. If the tumor is localized, meaning that it has not spread to other parts of the body, then surgery or radiation therapy may be an option. If the tumor has spread to other parts of the body, then chemotherapy or hormone therapy may be used.

If you have any questions about the pathology report, please talk to your doctor.

© 2024 Steimetz E et al. *JAMA Network Open*.

eFigure 2. Sample Exchange for Chatbot 2

JM

Please explain the following pathology report to someone without any high school education. Be concise:
"1. Prostate, left apex, biopsy:
- Benign prostatic tissue.
2. Prostate, left mid, biopsy:
- A minute focus of atypical small acinar proliferation (ASAP) in 1 out of 2 cores.
3. Prostate, left base, biopsy:
- Benign prostatic tissue.
4. Prostate, right apex, biopsy:
- Benign prostatic tissue.
5. Prostate, right mid, biopsy:
- A minute focus of atypical small acinar proliferation (ASAP) in 1 out of 2 cores.
- Focal chronic and acute inflammation.
6. Prostate, right base, biopsy:
- Benign prostatic tissue."

This report is about tests done on different parts of your prostate, which is a small gland that men have. It's like a doctor took a very tiny piece from six different spots on your prostate to look at under a microscope. In four of those spots, everything looked normal and healthy, like it should.
In two spots, they found a very small area where the cells are not behaving as they normally would. This doesn't mean you have cancer, but it means that they saw something unusual that they want to keep an eye on.

Also, in one of these two spots, they noticed some inflammation, which means that part of your prostate was a bit irritated or inflamed, like when you have a sore or a bruise. This could be because of an infection or just irritation.

In simple terms, they found something a little unusual, but it's not definitely bad news. The doctor will probably want to check you again in the future to make sure everything is okay.

JM

Respond in one word. Is the diagnosis benign or malignant?

Indeterminate.

JM

Choose one word to characterize the report. Your choices are normal, benign, atypical, pre-cancerous, malignant.

Atypical.

JM

What is the pathologic stage of the tumor?

The pathology report does not provide a stage for any tumor. It mentions "atypical small acinar proliferation (ASAP)" in two sites, which refers to abnormal cell growth. However, this is not a definitive diagnosis of cancer, so no stage can be assigned. Staging is typically done when a definite cancer diagnosis is made.

© 2024 Steimetz E et al. *JAMA Network Open*.
