# Supplementary material for: Use of Artificial Intelligence Chatbots in Interpretation of Pathology Reports
Source: JAMA Netw Open. 2024 May 22;7(5):e2412767. doi: 10.1001/jamanetworkopen.2024.12767 (PMC11112436; doi:10.1001/jamanetworkopen.2024.12767)
Supplement: Supplement 2. — Data Sharing Statement [file jamanetwopen-e2412767-s002.pdf]

Data Sharing Statement

Steimetz. Use of Artificial Intelligence Chatbots in Interpretation of Pathology Reports. *JAMA Netw Open*. Published May 22, 2024. doi:10.1001/jamanetworkopen.2024.12767

Data
Data available: No
